# Comparing the effects of dexmedetomidine versus propofol on the treatment of emergence agitation in adult patients after general anesthesia: study protocol for a randomized, superiority, controlled trial (DP-TEA Trial)

**DOI:** 10.1186/s13063-021-05743-2

**Published:** 2021-11-16

**Authors:** Zhaoyan Feng, Xiao Shi, Xue Yan, Yamin Zhu, Juan Gu, Hao Zhu, Weifeng Yu, Song Zhang

**Affiliations:** 1 Department of Anesthesiology, Renji Hospital, Shanghai Jiao Tong University School of Medicine, No. 160 Pujian Road, Shanghai, 200127 China; 2 Department of Anesthesiology, Shanghai Jiao Tong University Affiliated Sixth People’s Hospital, No. 600 Yishan Road, Shanghai, 200233 China

**Keywords:** Emergence agitation, Dexmedetomidine, Propofol, Adult patient

## Abstract

**Background:**

Emergence agitation (EA) after general anesthesia is a common complication in the post-anesthesia care unit (PACU). Once EA occurs, there are still no guidelines established for the treatment in adults. Propofol is excessively used in managing agitated patients in the PACU, but it lacks analgesia and can result in apnea. Intraoperative infusion of dexmedetomidine has been proven to have a preventive effect on EA, but the treatment effect of dexmedetomidine on EA remains unknown. This study aims to compare the effects between dexmedetomidine and propofol on relieving EA in adult patients after general anesthesia in the PACU.

**Methods:**

In this randomized, superiority, controlled clinical study, a total of 120 adult patients aged 18–65 years of both genders, with American Society of Anesthesiologists (ASA) classification I or II developing EA in the PACU after general anesthesia, will be enrolled. Patients will be randomized at a 1:1 ratio into two groups, receiving either a single dose of dexmedetomidine (0.7μg/kg) or propofol (0.5 mg/kg). The primary outcome is the proportion of patients having a recurrent EA within 15 min after intervention in the PACU.

**Discussion:**

Previous studies have focused on premedication for preventing EA, while therapeutics for reliving EA have rarely been reported. To our knowledge, this study is the first randomized, superiority, controlled trial to compare a bolus of dexmedetomidine with the current routine care for this indication.

**Trial registration:**

ClinicalTrials.govNCT04142840. Registered on October 26, 2019

**Supplementary Information:**

The online version contains supplementary material available at 10.1186/s13063-021-05743-2.

## Introduction

Emergence agitation (EA) from general anesthesia is a common complication in the daily clinical practice, with an incidence rate of 4.7 to 74% in adult patients undergoing various surgical procedures [1–6]. An agitated patient may remove the endotracheal tube, oxygen mask, catheters, or wound packing, leading to severe sequences such as hypoxia and hemorrhage. Furthermore, seriously agitated patients can constitute harmful behavior towards their care providers [7]. Despite the high prevalence and serious sequelae, the underlying mechanism of EA remains to be elusive. Several risk factors including premedication benzodiazepines, excruciating pain, long duration of surgery, breast- and abdominal-specific surgeries, presence of a tracheal tube and/or a urinary catheter, and preoperative anxiety have been proposed to be accountable for this phenomenon [2–4, 8].

In the past few years, anesthesiologists have been striving to reduce the incidence of EA and improve the quality of patient’s postoperative conditions with various drugs and techniques. Several drugs such as remifentanil [9], magnesium sulfate [5], and dexmedetomidine [1, 10, 11] have been suggested as latent prophylactic interventions of EA in adult patients. However, little progress has been made in therapeutics. According to our clinical experience, we can acknowledge that EA is still difficult to manage in agitated patients.

Nowadays, propofol is the most currently used drug in managing agitated patients, but it lacks analgesia and can suppress breathing transiently [12]. Dexmedetomidine is a highly selective α_2_-receptor agonist and has sympatholytic, analgesic, and sedative properties without causing respiratory depression at a clinically approved dosage [13]. Previous studies have shown the prophylactic effect of perioperative infusion of dexmedetomidine on EA. However, the treatment effects of dexmedetomidine on EA remain unknown.

This randomized study aims to compare the effects of dexmedetomidine and propofol on relieving EA in the post-anesthesia care unit (PACU) of adult patients after general anesthesia. Furthermore, we will also evaluate the effects of dexmedetomidine on patient’s quality of recovery 24 h after surgery. We hypothesize that dexmedetomidine is superior to propofol for relieving EA and improving the recovery quality in adult patients after general anesthesia.

## Objectives

The primary objective is to compare the effects of dexmedetomidine versus propofol on relieving EA after general anesthesia.

The secondary objectives include the following:

1. Comparing sedative outcomes after intervention between the two arms
2. Investigating the safety and tolerability of a single dose of 0.7μg/kg dexmedetomidine by monitoring the vital signs
3. Comparing the effects of dexmedetomidine versus propofol on postoperative nausea and vomiting (PONV), postoperative pain, and duration in the PACU
4. Evaluating the recovery quality between the two groups 24 h after surgery

## Method

### Study design and setting

**Fig. 1:**
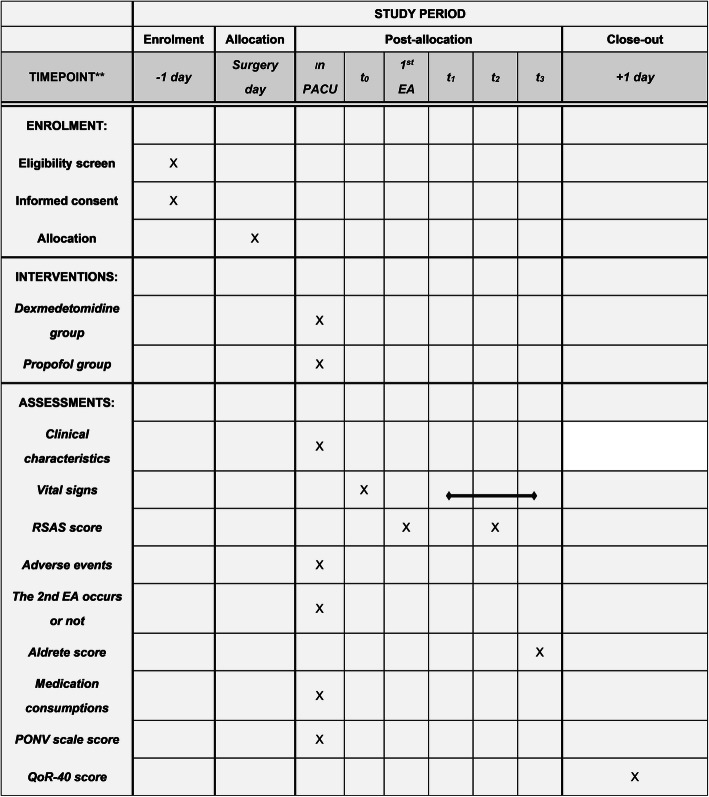
The schedule of enrollment, interventions, and assessments. RSAS, Riker’s Sedation-Agitation Scale; PONV, postoperative nausea and vomiting; QoR-40, quality-of-recovery questionnaire (including 40 items). *1^st^ EA, when first EA occurs; *t*_0_, when entering the PACU; *t*_1_, 1 min after the intervention; *t*_2_, 15 min after the intervention; *t*_3_, when leaving the PACU

This study is a randomized, superiority, controlled clinical trial, which is designed in accordance with the SPIRIT 2013 Checklist: recommended items to address in a clinical trial protocol (Additional file 1) [14]. The final study version 2.0 was approved by the Ethics Committee of Renji Hospital affiliated to Shanghai Jiao Tong University School of Medicine and registered in ClinicalTrials.gov. (NCT04142840). Time points of enrollment and assessments are shown in the Standard Protocol Items: Recommendations for Interventional Trials (SPIRIT) figure (Fig. 1). The study will be conducted in Renji Hospital affiliated to Shanghai Jiao Tong University School of Medicine from October 2019 to October 2021.

### Recruitment

A patient will be screened before the surgery day to ensure the potential eligibility for this research based on their laboratory tests and auxiliary examinations.

Clinical trial advertising will be posted in wards to introduce EA and the potential benefits of our experiment to the participants. Then, we will answer the patients’ queries during preoperative visits and enrollment.

### Consent

Due to their confusion and inability to lay down new memories, obtaining a signed consent from patients with EA is impossible. And obtaining consent immediately might lead to harmful delays to the initiation of treatment. Therefore, we will get verbal consent at the time of recruitment, with written consent taken as soon as possible after the intervention. More specifically, during preoperative anesthesia visit, the procedures involved in the study and the possible assignment will be explained briefly. Then, verbal consent from the patients or their legally authorized representatives will be gained by the study staff in the presence of an independent witness. Secondly, once EA happens, the patients will be randomly assigned into one of the two study groups to receive treatment immediately. Hereafter, the full written informed consents will be sought from the patients or their legally authorized representatives as soon as possible.

### Eligibility criteria

The inclusion criteria are adult patients aged 18–65 years of both genders, with American Society of Anesthesiologists (ASA) classification I or II, who develop EA after general anesthesia with verbal consent.

The exclusion criteria are as follows:

1. Age younger than 18 years or older than 65 years
2. ASA classification ≥ III
3. Preoperative lung dysfunction (including pneumonia, atelectasis, acute respiratory distress syndrome, acute lung injury)
4. Preoperative heart dysfunction (including severe cardiac coronary disease, unstable angina, left ventricular ejection fraction ≤ 30%, sick sinus syndrome, bradycardia: heart rate (HR) ≤ 50 beats/min, second- or third-degree atrioventricular block)
5. History of mental disease
6. Uncontrolled hypertension (baseline: systolic blood pressure (SBP) ≥ 160 mmHg or diastolic blood pressure (DBP) ≥ 110 mmHg)
7. Enrolled in other researches within 90 days
8. Allergic to dexmedetomidine or propofol
9. Body mass index (BMI) less than 18 kg/m^2^ or more than 30 kg/m^2^

### Outcomes

#### Primary outcomes

The primary outcome is the proportion of patients having a recurrent EA within 15 min after intervention in the PACU. EA is defined as a restless state with a Riker’s Sedation-Agitation Scale (RSAS) score of 5 or more (Table 1).

**Table 1:** Riker’s Sedation-Agitation Scale

7	Dangerous agitation	Pulling at tracheal tube, trying to remove catheters, climbing over the bed rail, striking at staff, thrashing from side to side
6	Very agitated	Does not calm down despite frequent verbal reminders of limits, require physical restraints, biting endotracheal tube
5	Agitated	Anxious or mildly agitated, attempting to sit up, calms down with verbal instructions
4	Non-agitated	Calm and cooperative
3	Sedated	Calm, awakens easily, follows commands, difficult to arouse, awakens to verbal stimuli or gentle shaking, but drifts off again, follows simple commands
2	Very sedated	Arouses to physical stimuli but does not communicate or follow commands, may move spontaneously
1	Unarousable	Minimal or no response to noxious stimuli, does not communicate or follow commands

#### Secondary outcomes

The following are the secondary outcomes:

1. RSAS scores when first EA occurs and at *t*_2_.
2. Vital signs at *t*_0_, *t*_1_, *t*_2_, and *t*_3_, including HR, mean arterial pressure (MAP), and peripheral oxygen saturation (SpO_2_).
3. Proportion of patients requiring rescue sufentanil during resuscitation in the PACU: an 11-point Numerical Rating Scale (NRS) is used to assess postoperative pain [15, 16] whenever patients ask for pain medication. If a NRS score is ≥ 5, an additional 5–10 μg sufentanil will be injected as a rescue medication.
4. Proportion of patients with PONV, evaluated by a 4-point PONV Scale (Table 2) during resuscitation in the PACU.
5. Time to discharge from the PACU.
6. Proportion of patients with adverse events after intervention in the PACU, including oxygen desaturation (defined as SpO_2_ < 90%, regarded as severe desaturation when SpO_2_ < 85%), severe bradycardia (defined as HR < 50 beats/min), shivering, dizziness, laryngospasm, severe coughing, and reintubation.
7. Recovering quality score assessed at 24 h after surgery using 40-item quality of recovery scale (QoR-40) (affiliated as Additional file 2).

**Table 2:** Postoperative nausea and vomiting scale

0	No nausea
1	Mild nausea
2	Sever nausea requiring antiemetics
3	Retching, vomiting, or both

### Randomization and blinding

An independent statistician from Renji Hospital Clinical Research Institute who is not involved in this trial will help us generate randomized numbers, and simple randomization will be used. Computer-generated random numbers will be concealed in opaque envelopes. A designated anesthesiologist who has received standardized training will evaluate a patient presented with a restless state to decide whether the patient is eligible for enrollment or not. Following enrollment, a designated PACU nurse will open the next sequential envelope containing a random number to assign the participant to either dexmedetomidine group (DEX group) or propofol group (PRO group) based on a 1:1 allocation ratio. Then, the unblinded PACU nurse will prepare and administer the study medication with one hand covering the syringe to ensure the assessor and care providers are unaware of the treatment allocation.

Participants, investigators, other healthcare providers, statisticians, and other study staff will be blinded to the assignment during this study. The PACU nurse administering the study medication will be the only unblinded individual involved in the study. In addition, this unblinded PACU nurse is not involved in assessing any study outcomes or other procedures related to the study.

### Sample size

Based on previous work on this topic [17], the recurrent rate of EA after the intervention by propofol was approximately 80%. A proposed EA recurrence rate for patients after receiving dexmedetomidine is 50%. We used the following formulas for sample size calculation (to test whether the proportion in group “A,” *p*_*A*_, is superior to the proportion in group “B,” *p*_*B*_):

*n*_*A*_ = *kn*_*B*_ and $$ {n}_B=\left(\frac{p_A\left(1-{p}_A\right)}{k}+{p}_B\left(1-{p}_B\right)\right){\left(\frac{z_{1-\alpha /2}+{z}_{1-\beta }}{p_A-{p}_B-\delta}\right)}^2 $$

Here, we defined *α* = 0.05 (two-sided), *β* = 0.2, *p*_*A*_ = 0.5, *p*_*B*_ = 0.8, *δ* is the superiority margin (*δ* was set as − 0.05 in this trial), and *k* is the ratio between the sample sizes of the two groups (*k* = 1). We also considered a 10% dropout rate, so we finally decided to enroll 60 patients per group.

### Intervention

Electrocardiogram (ECG), SpO_2_, HR, and non-invasive blood pressure (NBP) will be monitored once the eligible patient enters the operating room at 5-min intervals. General anesthesia will be administered with midazolam 0.05 mg/kg, propofol 2–2.5 mg/kg, and sufentanil 0.2–0.5 μg/kg, and tracheal intubation will be facilitated with rocuronium 0.7 mg/kg. After intubation, anesthesia will be maintained with sevoflurane and remifentanil, according to the guidelines of bispectral index (BIS) target ranging between 40 and 60. The neuromuscular blockade will be maintained via intermittent boluses of rocuronium. End-tidal carbon dioxide will be controlled between 35 and 45 mmHg. BP will be kept between 80 and 120% of the baseline by vasoactive agents or adapting anesthesia depth. HR will be maintained between 50 and 100 beats/min with atropine and esmolol.

**Fig. 2:**
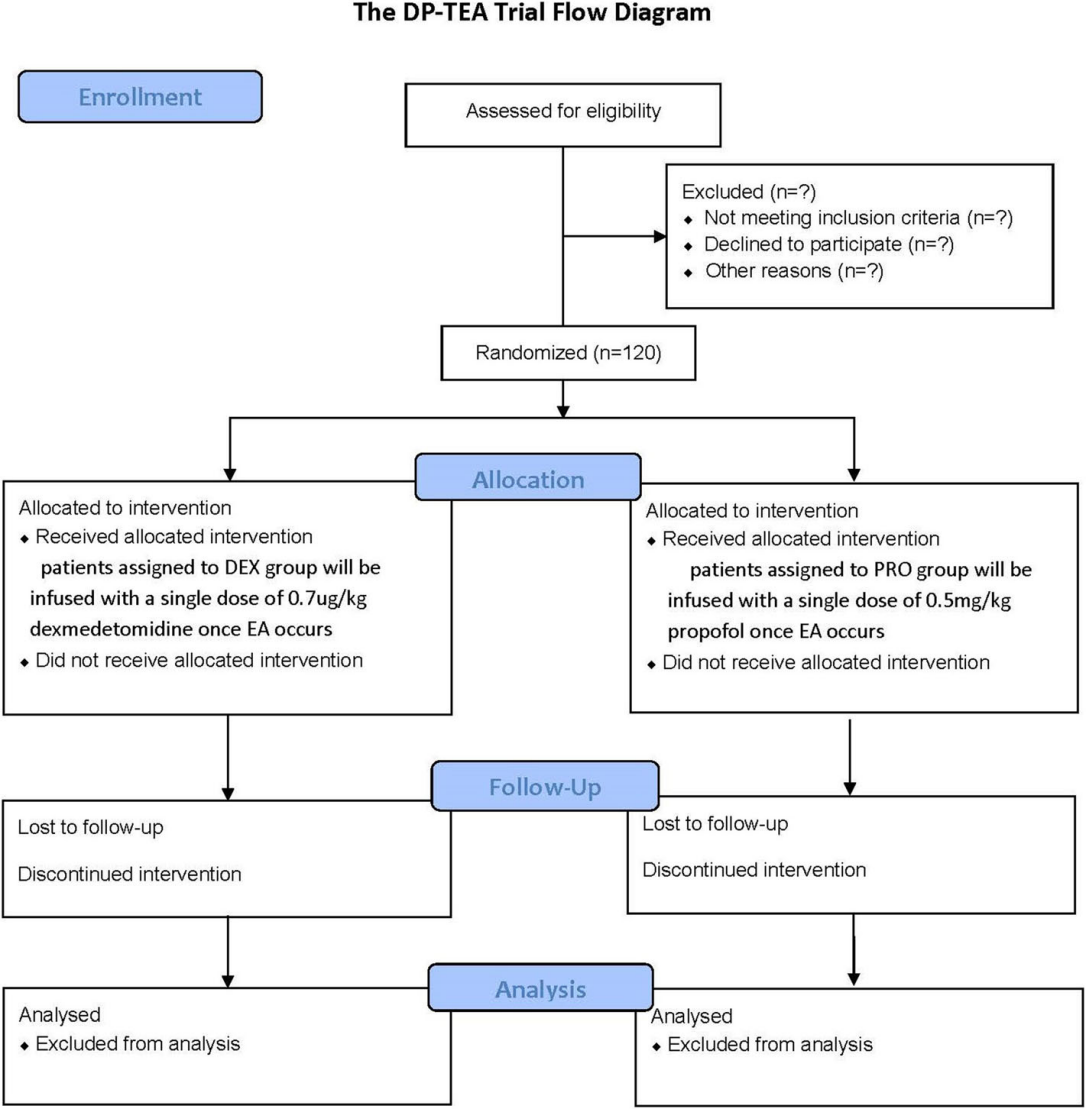
The flowchart of the trial

By consulting the surgeons, all the anesthesia agents will be discontinued 5 min prior to the end of the surgery, and the patient will be transferred to the PACU. ECG, HR, SpO_2_, and NBP are monitored immediately and then measured at 5-min intervals. Reversal agents (neostigmine 0.04 mg/kg and atropine 0.15 mg/kg) are given to antagonize the residual muscular relaxant when patients exhibit spontaneous breathing and a return of two visual twitch responses in the train-of-four (TOF) stimuli. Extubation will be performed when the patient can respond to commands and tidal volume is more than 5 ml/kg with regular breathing. Patients presented with a restless state will be assessed in the PACU by a designated anesthesiologist who has received standardized training. The flowchart is shown in Fig. 2.

Once EA occurs, the RSAS scores of agitated patients will be recorded. Patients assigned to the DEX group will be infused with 0.7μg/kg dexmedetomidine, and PRO group patients are to be treated with 0.5 mg/kg propofol by the unblinded PACU nurse. Hemodynamic parameters, including HR, MAP, and SpO_2_, are recorded at *t*_0_, *t*_1_, *t*_2_, and *t*_3_. Whether the second EA occurs or not within the following 15 min after the intervention will be noted, and the RSAS scores at 15 min after intervention (*t*_2_) will also be recorded. Postoperative pain and PONV will be assessed, while sufentanil and ondansetron will be used as the rescue medications. The patient will be transferred to the ward once meeting an Aldrete score ≥ 9 [18]. In addition, adverse events and length of PACU stay will also be recorded.

After discharge, patients will be followed up at 24 h with a QoR-40 scale to be finished [19].

### Data collection and management

Preoperative variables are as follows: demographic characteristics (name, age, sex, height, weight, BMI), diagnosis, scheduled surgery procedures, ASA classification, laboratory results (liver and kidney function tests, routine blood tests), and an ECG report.

Perioperative data include the duration of the surgery, the dosage of medications used in induction and maintenance of general anesthesia, and the fluid input and output.

Postoperative parameters include vital signs (HR, MAP, and SpO_2_), the RSAS scores, medication consumption, adverse events, the PACU duration, and the QoR-40 scale scores 24 h after surgery.

All data will be recorded into a CRF timely, entirely, and correctly. The CRF will be signed by the supervisor and transferred to clinical data coordinators for data management and entry. To ensure the accuracy of the data, two clinical data coordinators should independently enter and proofread double copies. Data will be presented with “A group” or “B group” on CRFs or in our database. Data queries generated by the data manager should be answered by the investigator timely, so that data modification, confirmation, and entry can be performed efficiently.

The database will be locked after conformation of the completeness, reliability, and accuracy of all data. Only the principal investigator, the database manager, or a statistician responsible for the statistical analysis have access to the database.

### Data monitoring and auditing

Data monitors from Renji Hospital Clinical Research Institute are independent of this trial and are authorized to verify the accuracy and integrity of data and the conduct of the trial in compliance with the currently approved protocol. Auditors are also from Renji Hospital Clinical Research Institute and independent of this clinical trial, to evaluate trial conduct and compliance with the protocol, Standard Operating Procedures (SOPs), Good Clinical Practice (GCP), and other applicable regulatory requirements.

### Confidentiality

All related information of participants will be kept in a locked file cabinet, which is only available to researchers to ensure that the research is conducted in accordance with the regulations. Members of the government management department or ethics review committee can access the patients’ data in the research unit as required. When this research result is published, no personal information will be disclosed.

### Data analysis

Statistical analysis will be performed using the SPSS software (version 24.0; SPSS Inc., IBM, Chicago, IL, USA). Continuous variables (such as age, height, weight, BMI, duration of the surgery and anesthesia, length of PACU stay) will be presented as mean ± standard deviation (*SD*) or median (interquartile range) based on normal distribution checked by the Kolmogorov-Smirnov test. The differences between the groups will be analyzed with an independent *t*-test or Mann–Whitney *U* test as appropriate. Categorical variables (such as the recurrence rate of EA, the incidence adverse events) will be expressed as number of patients (percentage) and analyzed using the chi-squared test or Fisher’s exact test. Repeated-measures data like vital signs will be analyzed by two-way analysis of variance (ANOVA) followed by Bonferroni correction. A two-tailed *P* value < 0.05 is considered to be statistically significant.

As all of our endpoints will be identified in the PACU or 24 h after surgery, and data recorded on CRF will be double-checked, small amount of missing data will be expected in this trial. To accomplish a completed case analysis, multiple imputations will be undertaken in each group separately with a sensitivity analysis for the assumptions performed (i.e., missing completely at random). Full details of the imputation procedure will be reported.

### Analysis population

We will assess and report all outcomes as “intention-to-treat” analysis including all randomized patients meeting the inclusion criteria and not fulfilling the exclusion criteria with consent to participate. In addition, we will exclude patients with major protocol violations defined as failure to receive the total drug dose or failure to complete follow-up in a supplemental per-protocol analysis.

### Safety consideration

In our study, both dexmedetomidine and propofol are common anesthetics in clinical anesthesia. A rapid bolus of dexmedetomidine (0.5 μg/kg) has been proven to be hemodynamically acceptable in children [20], and propofol with a dose of 0.5 mg/kg is a routine usage. To ensure patients’ safety, firstly, an unblinded nurse will implement the infusion; secondly, rescue medication like vasoactive agents and non-invasive positive pressure ventilators will be prepared; and thirdly, participants will be continuously monitored until 24 h after surgery.

### Adverse event reporting

Adverse events (AEs) are any adverse symptoms, abnormal signs, and abnormal laboratory results after applying intervention measures, regardless of whether there is a causal relationship with the drug. AEs will be timely recorded and treated promptly according to routine practice and should be followed up until it has completely resolved. The documentation of AEs should be in detail, including time of occurrence, diagnosis, time of diagnosis, management, duration of persistence, and sequelae.

In our trial, AEs related to a bolus of dexmedetomidine include but not limited to bradycardia, hypotension/hypertension, nausea, vomiting, and xerostomia. And AEs related to a bolus of propofol include but not limited to apnea, bradycardia/tachycardia, hypotension, injection site reactions (burning, stinging, or pain), and dizziness. Apart from the expected AEs above, a systematic approach is used to classify and collect other unexpected AEs considering the correlation between AEs and intervention and the severity criterion.

The correlation between adverse events and study treatment is divided into five levels: definitely related, probably related, possibly related, possibly unrelated, and definitely unrelated.

The severity criteria are as follows:

*Mild*: signs and symptoms are mild and transient, do not affect daily activity, do not need treatment, and usually recover after rest.

*Moderate*: signs and symptoms last longer, mildly affect daily activity, and recover after simple treatment.

*Severe*: signs and symptoms last even longer, significantly affect daily activity and life, and do not recover after simple treatment.

Once a severe AE occurs, the anesthesiologist in the PACU responsible for assessing participants can request unmasking of the allocation or interrupting/adjusting the infusion of the drug. The documentation of unmasking should be in detail. Severe AEs will be reported to the Ethics Committee of Renji Hospital within 24 h in a written report. In case of study drug-related death, we will immediately stop the clinical trial and report the event to the Ethics Committee as soon as possible. The related documents will be recorded in detail and carefully preserved. The Ethics Committee will decide to restart the study. If the patient’s harm level meets the insurance claims, payment will be arranged as soon as possible.

## Discussion

Previous studies about EAs mainly focused on the preventive strategies [1, 4, 11, 21], and little progress has been made in the therapeutics of EA. Once occurring without immediate and effective treatment, EA will add an extra burden to nursing work in the PACU and bring negative effects to the patients’ prognosis. Therefore, it is of great necessity to find a more ideal medicine for the management of EA with as few side effects as possible. Based on the prophylactic role of dexmedetomidine in EA and successful case reports of using dexmedetomidine as rescue therapy [22] for emergence delirium in adults reported previously, we designed this clinical trial to compare dexmedetomidine to propofol, which is a major choice in terms of EA management in China nowadays, to provide the evidence of the potential superiority of dexmedetomidine on improving the recovering quality of agitated adult patients after general anesthesia.

One major concern of our trial is that we need to seriously control medication during general anesthesia in each arm to minimize the residual effects of other medications except propofol and dexmedetomidine on resuscitation. However, there are also limitations in our study. Firstly, to promise the patients’ safety, our staff cannot be blinded to the two medications. But the unblinded staff would not be involved in the assessment and data analysis. Secondly, as a single-center trial, our results may have limited external validity and are not necessarily generalizable to a broader population. Thus, physicians should apply the findings of this trial only after careful evaluation of their methodology. Thirdly, selection bias may exist, but the bias can be reduced maximally since our hospital is a top-level general hospital in China where various surgeries of different organs and systems are involved.

## Trial status

The recruitment of this trial was initiated on November 1, 2019, but suspended for 6 months since January 18, 2020, due to the COVID-19 pandemic. We restarted the trial on July 25, 2020, and are currently enrolling patients. We expect to complete the study on December 31, 2021.

Protocol version number: DP-TEA 2.0, September 10, 2019.

Original version: DP-TEA 1.0, July 13, 2019

Amendment number: 1

Primary reason for amendment: elaboration of blinding procedure

Primary changes: In protocol 1.0 (July 13, 2019), we simply stated “our trial is blinded to patients, staff responsible for the outcome assessment, data collectors, and statisticians.” In protocol 2.0 (September 10, 2019), we added details about how to blind as required by the Ethics Committee.

## Supplementary Information

**Additional file 1:.** The SPIRIT 2013 Checklist: Recommended items to address in a clinical trial protocol.

**Additional file 2:.** Qo-R 40 scale.

**Additional file 3:.** A supplementary table with all items from the WHO trial registry data set.

**Additional file 4:.** Informed consent form.

## Data Availability

The data that support the findings of this study will be available from the corresponding authors upon reasonable request.

## Abbreviations

EA: Emergence agitation
PACU: Post-anesthesia care unit
ASA: American Society of Anesthesiologists
BMI: Body mass index
QoR-40: Quality-of-Recovery Questionnaire
RSAS: Riker Sedation-Agitation Scale
SBP: Systolic blood pressure
DBP: Diastolic blood pressure
PONV: Postoperative nausea and vomiting
NRS: Numerical Rating Scale
MAP: Mean arterial pressure
BIS: Bispectral index
TOF: Train-of-four
SpO_2_: Peripheral oxygen saturation
SOP: Standard operating procedure
GCP: Good Clinical Practice
AEs: Adverse effects
