# Supplementary material for: Comparing the effects of dexmedetomidine versus propofol on the treatment of emergence agitation in adult patients after general anesthesia: study protocol for a randomized, superiority, controlled trial (DP-TEA Trial)
Source: Trials. 2021 Nov 16;22:811. doi: 10.1186/s13063-021-05743-2 (PMC8593636; doi:10.1186/s13063-021-05743-2)
Supplement: Supplementary file 2 — Additional file 2:. Qo-R 40 scale. [file 13063_2021_5743_MOESM2_ESM.pdf]

Date: _ _ / _ _ _ / _

Patient Survey (QoR – 40)

Name: ____________________ study #: ________ Hospital UR #: ___________

PART A

How have you been feeling in the last 24 hours?

(1 to 5, where : 1 = very poor and 5 = excellent)

For example: If you have been able to breathe easily all of the time, you should indicate this by circling the response 5 = ***all of the time*** as shown below:

	None of the time	Some of the time	Usually	Most of the time	All of the time
Able to breathe easily	1	2	3	4	(5)

	None of the time	Some of the time	Usually	Most of the time	All of the time
**Comfort**					
Able to breathe easily	1	2	3	4	5
Have had a good sleep	1	2	3	4	5
Been able to enjoy food	1	2	3	4	5
Feel rested	1	2	3	4	5
**Emotions**					
Having a feeling of general well-being	1	2	3	4	5
Feeling in control	1	2	3	4	5
Feeling comfortable	1	2	3	4	5

How have you been feeling in the last 24 hours?

(1 to 5, where : 1 = very poor and 5 = excellent)

	None of the time	Some of the time	Usually	Most of the time	All of the time
**Physical Independence**					
Have normal speech	1	2	3	4	5
Able to wash, brush teeth or shave	1	2	3	4	5
Able to look after your own appearance	1	2	3	4	5
Able to write	1	2	3	4	5
Able to return to work or usual home activities	1	2	3	4	5
**Patient Support**					
Able to communicate with hospital staff (when in hospital)	1	2	3	4	5
Able to communicate with family or friends	1	2	3	4	5
Getting support from hospital doctors (when in hospital)	1	2	3	4	5
Getting support from hospital nurses (when in hospital)	1	2	3	4	5
Having support from family or friends	1	2	3	4	5
Able to understand instructions and advice	1	2	3	4	5

PART B

Have you had any of the following in the last 24 hours?

(5 to 1, where: 5 = excellent and 1 = very poor)

	None of the time	Some of the time	Usually	Most of the time	All of the time
**Comfort**					
Nausea	5	4	3	2	1
Vomiting	5	4	3	2	1
Dry-retching	5	4	3	2	1
Feeling restless	5	4	3	2	1
Shaking or twitching	5	4	3	2	1
Shivering	5	4	3	2	1
Feeling too cold	5	4	3	2	1
Feeling dizzy	5	4	3	2	1
**Emotions**					
Had bad dreams	5	4	3	2	1
Feeling anxious	5	4	3	2	1
Feeling angry	5	4	3	2	1
Feeling depressed	5	4	3	2	1
Feeling alone	5	4	3	2	1
Had difficulty falling asleep	5	4	3	2	1

Have you had any of the following in the last 24 hours?

(5 to 1, where: 5 = excellent and 1 = very poor)

	None of the time	Some of the time	Usually	Most of the time	All of the time
**Patient Support**					
Feeling confused	5	4	3	2	1
**Pain**					
Moderate pain	5	4	3	2	1
Severe pain	5	4	3	2	1
Headache	5	4	3	2	1
Muscle pains	5	4	3	2	1
Backache	5	4	3	2	1
Sore throat	5	4	3	2	1
Sore mouth	5	4	3	2	1

Thank you for your assistance.

Please check that all questions have been answered.

If you have any questions, please contact: Jenny Hunt or Helen Fletcher through the hospital's switchboard (03) 9276 2000.
