# Supplementary material for: Comparing the effects of dexmedetomidine versus propofol on the treatment of emergence agitation in adult patients after general anesthesia: study protocol for a randomized, superiority, controlled trial (DP-TEA Trial)
Source: Trials. 2021 Nov 16;22:811. doi: 10.1186/s13063-021-05743-2 (PMC8593636; doi:10.1186/s13063-021-05743-2)
Supplement: Supplementary file 3 — Additional file 3:. A supplementary table with all items from the WHO trial registry data set. [file 13063_2021_5743_MOESM3_ESM.pdf]

All items from the World Health Organization Trial Registration Data Set (SPIRIT item 2b)

**Data category**	**Information**
Primary registry and trial identifying number	ClinicalTrials.gov<br>NCT04142840
Date of registration in primary registry	26 October 2019
Secondary identifying numbers	DP-TEA 2.0
Source(s) of monetary or material support	Youth Program of National Natural Science Foundation of China (No. 81801096, to Song Zhang)
Primary sponsor	Renji Hospital
Secondary sponsor(s)	/
Contact for public queries	Weifeng Yu, MD, PhD,86+13901961704, yuweifeng@renji.com
Contact for scientific queries	Song Zhang, MD, PhD, 86+15150012530, zhangsong@renji.com
Public title	Comparing the Effects of Dexmedetomidine versus Propofol on the Treatment of Emergence Agitation in Adult Patients after General Anesthesia
Scientific title	Comparing the Effects of Dexmedetomidine versus Propofol on the Treatment of Emergence Agitation in Adult Patients after General Anesthesia: study protocol for a randomized controlled trial (DP-TEA Trial)
Countries of recruitment	China
Health condition(s) or problem(s) studied	Emergence agitation after general anesthesia
Intervention(s)	***Intervention Name:*** Dexmedetomidine group<br>***Intervention Description:*** Once emergence agitation occurs, the participant assigned to dexmedetomidine group will be infused with a single dose of 0.7ug/kg dexmedetomidine.<br><br>***Intervention Name:*** Propofol group<br>***Intervention Description:*** Once emergence agitation occurs, the participant assigned to propofol group will be infused with a single dose of 0.5mg/kg propofol.

Data category	Information
Key inclusion and exclusion criteria	***Inclusion criteria:***<br>• adult patients aged 18-65 years of both genders, with American Society of Anesthesiologists (ASA) classification I or II developing EA after general anesthesia with verbal consent.<br>***Exclusion Criteria:***<br>• age younger than 18 years or older than 65 years;<br>• ASA classification ≥III;<br>• preoperative lung dysfunction (including pneumonia, atelectasis, acute respiratory distress syndrome, acute lung injury, and so on);<br>• preoperative heart dysfunction (including sever cardiac coronary disease, unstable angina, left ventricular ejection fraction≤30％, sick sinus syndrome, bradycardia: heart rate (HR)≤50beats/min, the second or third degree of atrioventricular block);<br>• history of mental disease;<br>• uncontrolled hypertension (baseline: systolic blood pressure (SBP)≥160mmHg or diastolic blood pressure (DBP)≥110mmHg);<br>• enrolled in other researches within 90 days;<br>• allergic to dexmedetomidine or propofol;<br>• 10) body mass index (BMI) less than 18 kg/m^2 or more than 30 kg/m^2.
Study type	Interventional<br>***Allocation:*** randomized<br>***Intervention model:*** parallel<br>***Masking:*** Blind to patients, outcome assessors, care providers, data collectors and statisticians.<br>***Primary purpose:*** recovery quality after general anesthesia research
Date of first enrolment	1 November 2019
Target sample size	120
Recruitment status	Enrolling
Primary outcome(s)	The primary outcome is the proportion of patients having a recurrent EA within 15min after intervention in the PACU. EA is defined as a restless state with a Riker’s Sedation-Agitation Scale (RSAS) score of 5 or more

Key secondary outcomes	• RSAS scores when first EA occurs and at t_2;<br>• Vital signs at t_0, t_1, t_2, t_3, including HR, MBP and SpO_2;<br>• Proportion of patients requiring rescue sufentanil during resuscitation in the PACU: an 11-point numerical rating scale (NRS) is used to assess postoperative pain whenever patients ask for pain medication. If a NRS score is≥5, additional 5-10μg sufentanil will be injected as rescue medication.<br>• Proportion of patients with PONV, evaluated by a four-point PONV Scale during resuscitation in the PACU;<br>• Time to discharge from the PACU;<br>• Proportion of patients with adverse events after intervention in the PACU, including oxygen desaturation (defined as SpO2<90%, regarded as severe desaturation when SpO2<85%), severe bradycardia (defined as HR<50 beats/min), shivering, dizziness, laryngospasm, severe coughing, and reintubation;<br>• Recovering quality score assessed at 24 hours after surgery using 40-item quality of recovery scale (QoR-40)
Ethics Review	• Status: Approved<br>• Date of approval: 14th, October 2020<br>• Name and contact details of Ethics committee(s): the Ethics committee of Renji Hospital affiliated to Shanghai Jiao Tong University School of Medicine
Completion date	• We expect to complete the study on December 31, 2021.
Summary Results	• /
IPD sharing statement	• The data that support the findings of this study will be available from the corresponding author upon reasonable request.
