# Supplementary material for: Comparing the effects of dexmedetomidine versus propofol on the treatment of emergence agitation in adult patients after general anesthesia: study protocol for a randomized, superiority, controlled trial (DP-TEA Trial)
Source: Trials. 2021 Nov 16;22:811. doi: 10.1186/s13063-021-05743-2 (PMC8593636; doi:10.1186/s13063-021-05743-2)
Supplement: Supplementary file 4 — Additional file 4:. Informed consent form. [file 13063_2021_5743_MOESM4_ESM.pdf]

Program name: Comparing the Effects of Dexmedetomidine versus Propofol on the Treatment of Emergence Agitation in Adult Patients after General Anesthesia (DP-TEA Trial)
Informed consent version number and version date: Consent 1.0/20190713

Research institute: Renji Hospital affiliated to Shanghai Jiao Tong University School of Medicine
Main researcher: Dr. Song Zhang

Dear patient:
We sincerely invite you to participate in a clinical study.
First, please read this informed consent form as carefully as possible, as it can help you decide whether to join the study or not. The following content includes why the study should be conducted, the study procedure and duration, and the benefits, risks, and discomforts that may be brought to you after participating in the study.
You can also discuss with your relatives and friends if you want or ask the doctor to explain and help you make a decision.
Your participation in this study is voluntary, and this research plan has been reviewed by the institution's ethics committee.

Background
Emergence agitation (EA) from general anesthesia is a common complication in the post-anesthesia care unit (PACU), with an incidence of 4.7% to 74% in adult patients undergoing various surgical procedure. An agitated patient may remove endotracheal tube, oxygen masks, catheter or wound packing, leading to serious sequences such as hypoxia and hemorrhage. Dexmedetomidine is currently a widely used auxiliary drug for general anesthesia. Clinical studies have proved that dexmedetomidine has a certain preventive effect on postoperative agitation. However, the therapeutic effect of dexmedetomidine for EA is still lacking of prospective clinical evidence. This study intends to compare the therapeutic effect of dexmedetomidine and propofol on EA during the recovery period after general anesthesia and the effects of two drugs on the quality of resuscitation.

Purpose
This research group intends to conduct a randomized controlled clinical study to investigate whether the treatment effect of dexmedetomidine on EA can improve the quality of postoperative recovery for agitated patients, so as to provide patients with safer and more comfortable recovery procedure.

Procedure
If you are willing to participate in this study, our doctors will：ask and record your medical history, review your test report, assess the baseline, and determine whether you can be enrolled before surgery. Then you will be randomly divided into a

propofol group (P) and a dexmedetomidine group (D). If EA happens to you after you have undergone general anesthesia, propofol or dexmedetomidine will be administrated. This part of the operation does not require your extra cooperation. What you need to cooperate with us is that we will ask you to resume the quality survey within the next day after the operation. You can voluntarily participate in the study and sign an informed consent form.

Alternative treatment

Participating in this study may or may not improve your health, you can choose:

1.Continue your routine treatment without participating in this study. The conventional treatment is: propofol.

2. No treatment.

Please discuss your decision with your doctor.

Possible adverse reactions

Possible adverse reactions of dexmedetomidine are hypotension, bradycardia, dry mouth, etc. We will closely monitor the whole process, and will promptly give appropriate treatment in case of adverse reactions.

Anticipated profits

Both propofol and dexmedetomidine are provided free of charge. If dexmedetomidine is used for EA during the general anesthesia recovery period, the quality of your perioperative recovery may be improved; the length of hospitalization and hospitalization costs will be reduced; and your recovery after surgery will be more stable and comfortable.

Compensation

Since this study is completed during hospitalization and do not incur additional transportation costs, there is no direct financial compensation. If you get injured by participating in this research, you can get corresponding financial compensation based on the appraisal of the relevant department and consultation with the patient and family members.

Free treatment

During the study, you do not need to pay for the drugs and consumables needed for EA during the general anesthesia recovery period. We will be responsible for all your expenses in this regard. If you are injured by participating in this study, you can get free treatment provided by the anesthesiology department.

Confidentiality

If you decide to participate in this study, your participation in the trial and the personal data in the trial are confidential. The research physician and other researchers will use your medical information to conduct research. This information may include your name, address, telephone number, medical history, and information obtained

during your research visit. Your file will be kept in a locked file cabinet, which is only available to researchers. To ensure that the research is conducted in accordance with the regulations, members of the government management department or ethics review committee can access your personal data in the research unit as required. When this research result is published, no personal information about you will be disclosed.

Voluntary

Whether to participate in the study or not entirely depends on your wishes. You can refuse to participate in this study or withdraw from this study at any time during the study. This will neither affect the relationship between you and the doctor, nor will it affect the loss of your medical or other interests. Your data will then not be included in the study results, and any of your medical treatment and rights will not be affected by this.

If you need other treatments, or you do not follow the research plan, for the best interests of you, the doctor or investigator may stop you from continuing to participate in the research at any time during the research process.

Contact

If you have questions related to this study or any discomfort/injury during the study, you can contact Dr. Song Zhang at 021-668383702 or 15150012530.

If you have any questions or complain about the researchers during the research process, or have questions about the rights and interests of the participants in this study, you can contact the Ethics Committee of Renji Hospital through 021-6683833364.

Subject's consent statement:

I have read this informed consent.

I have the opportunity to ask questions and all questions have been answered.

I understand that participation in this study is voluntary.

I can choose not to participate in this research, or I will notify the investigator at any time and withdraw without discrimination or retaliation, and any of my medical treatment and rights will not be affected.

If I need other treatment, or if I do not follow the research plan, or if there is an injury related to the research, or for any other reason, the research physician can terminate my continued participation in this research.

I will receive a signed copy of the "informed consent".

Signature of subject or family members:__________________________

Relationship with subjects:__________________________

date：__________________________

Tel：__________________________

（*：If the subject is incapacitated or restricted, a legal representative is required.）

Signature of legal representative：__________________________

date：__________________________

Tel：__________________________

Note: This page is the subject's signature page. The research doctor will explain the research content and related information to the subject in detail. If the subject has doubts about the content of the study, the researcher should immediately explain them specifically. After completing this form, both the researcher and the subject should keep an original document.
